# Actinomycins from Soil-Inhabiting *Streptomyces* as Sources of Antibacterial Pigments for Silk Dyeing

**DOI:** 10.3390/molecules28165949

**Published:** 2023-08-08

**Authors:** Tananya Nuanjohn, Nungruthai Suphrom, Nareeluk Nakaew, Wasu Pathom-Aree, Nattha Pensupa, Apiradee Siangsuepchart, Bernard Dell, Juangjun Jumpathong

**Affiliations:** 1 Department of Agricultural Science, Faculty of Agriculture, Natural Resources and Environment, Naresuan University, Phitsanulok 65000, Thailand; 2 Department of Chemistry, Faculty of Science, Center of Excellence for Innovation in Chemistry, Naresuan University, Phitsanulok 65000, Thailand; 3 Department of Microbiology and Parasitology, Faculty of Medical Science, Naresuan University, Phitsanulok 65000, Thailand; 4 Research Center of Excellence in Bioresources for Agriculture, Industry and Medicine, Department of Biology, Faculty of Science, Chiang Mai University, Chiang Mai 50200, Thailand; 5 Department of Agro-Industry, Faculty of Agriculture, Natural Resources and Environment, Naresuan University, Phitsanulok 65000, Thailand; 6 Department of Agro-Industrial Biotechnology, Maejo University Phrae Campus, Phrae 54140, Thailand; 7 School of Agricultural Sciences, Murdoch University, Perth 6150, Australia; 8 Center of Excellence in Research for Agricultural Biotechnology, Faculty of Agriculture, Natural Resources and Environment, Naresuan University, Phitsanulok 65000, Thailand

**Keywords:** actinomycins, antibacterial pigment, bioassay-guided fractionation, *Streptomyces*

## Abstract

Actinobacteria produce a broad spectrum of bioactive substances that are used in the pharmaceutical, agricultural, and biotechnology industries. This study investigates the production of bioactive substances in *Streptomyces*, isolated from soil under five tropical plants, focusing on their potential as natural antibacterial dyes for silk fabrics. Out of 194 isolates, 44 produced pigments on broken rice as a solid substrate culture. Eight antibacterial pigmented isolates from under *Magnolia baillonii* (TBRC 15924, TBRC 15927, TBRC 15931), *Magnolia rajaniana* (TBRC 15925, TBRC 15926, TBRC 15928, TBRC 15930), and *Cinnamomum parthenoxylon* (TBRC 15929) were studied in more detail. TBRC 15927 was the only isolate where all the crude extracts inhibited the growth of the test organisms, *Staphylococcus epidermidis* TISTR 518 and *S. aureus* DMST 4745. The bioactive compounds present in TBRC 15927 were identified through LC-MS/MS analysis as belonging to the actinomycin group, actinomycin D (or X_1_), X_2_, and X_0β_. Also, the ethyl acetate crude extract exhibited non-toxicity at an IC_50_ value of 0.029 ± 0.008 µg/mL on the mouse fibroblast L-929 assay. From the 16S rRNA gene sequence analysis, TBRC 15927 had 100% identity with *Streptomyces gramineus* JR-43^T^. Raw silk dyed with the positive antimicrobial TBRC 15927 extract (8.35 mg/mL) had significant (>99.99%) antibacterial properties. *Streptomyces gramineus* TBRC 15927 is the first actinomycin-producing strain reported to grow on broken rice and shows promise for antibacterial silk dyeing.

## 1. Introduction

Pigments play a crucial role in many life forms on Earth. Humans use pigments to enhance food and as a dye for various natural products. Synthetic dyes were introduced in 1856, and this led to a decrease in the use of natural-colored compounds [1]. However, as some synthetic dyes are unhealthy and toxic to the environment, the demand for natural dyes and pigments has increased in recent years [2]. This has increased research interest in natural colors for the food, cosmetic, and textile industries [3].

Soil actinobacteria are an important source of bioactive substances, and these include antibiotics, anticancer, and antioxidants [4]. Among the actinobacteria, *Streptomyces* is well known for being a rich source of antibiotics and other functional compounds that can be used in medicine and agriculture [5,6]. Moreover, some *Streptomyces* can produce pigments to protect their cells from environmental stress [7,8]. As a result, pigment-producing strains can be a useful alternative to synthetic coloring agents in the food, cosmetics, and textile industries.

Numerous studies have revealed that soil-inhabiting *Streptomyces* play an essential role in rhizosphere bioactivity, but few studies have explored their antibacterial pigments [9,10,11,12]. The genus *Streptomyces* is a rich source of diverse pigments, including: actinorhodin, a red–blue pigment with antibacterial activity produced by *Streptomyces coelicolor* [13]; prodigiosin (red pigment) isolated from *Streptomyces* sp. strain NP4 [14]; and dark melanoid compounds produced by *Streptomyces glaucescens* NEAE-H [15]. Furthermore, extracts of red–orange–yellow pigments obtained from a *Streptomyces* sp. were often found to contain actinomycins [16,17,18,19]. Due to the lower health risk of microbial colorants, there is interest in exploiting natural pigments for dyeing fabrics [14,20].

Actinomycins are well-known polypeptide antibiotics that have been isolated from *Streptomyces* [21,22,23] and *Nocardiopsis* [24]. The actinomycin-producing *Streptomyces antibioticus* (previously known as *Actinomyces antibioticus*) was first reported to form a dark brown to black pigment on protein- and peptone-containing media [25]. More than 41 actinomycin structures were isolated [26], with actinomycin D being extensively used in research due to its ability to intercalate with DNA and thereby inhibit the progression of RNA polymerases [27,28]. Several studies examined the antimicrobial, antitumor, and anti-tuberculosis activities of the new actinomycin producers [19,29,30]. Actinomycin L_1_ and L_2_ were recently isolated from *Streptomyces* sp. MBT27 and possess potent antibacterial activity against Gram-positive bacteria [31]. Actinomycin X_2_ was used in the application of a natural pigment for silk fabric [20] and immobilized silk fibroin film [32] in addition to actinomycin D, which is the most studied compound.

The application of microbial pigments in textile dyeing necessitates the careful orchestration of multiple steps. The process initiates with the dissolution of the pigment into a solution, a procedure that can be accomplished by employing solvents such as ethanol, methanol, or acetone within the dye bath. During the dyeing phase, several factors, such as the pH, temperature, immersion duration, and pigment concentration, must be systematically regulated. The pH of the solution is contingent upon the nature of the textile material; cellulose-based substances require a basic pH environment, while protein-based fibers, including wool and silk, necessitate an acidic environment for successful dyeing. The temperature of the dye bath, a critical variable, directly influences the dyeing rate and color depth. Elevated temperatures can potentially abbreviate the immersion time needed to reach a comparable color depth. Simultaneously, the concentration of the pigment can significantly influence the color depth and the degree of dye exhaustion. Notably, the pigments may exhibit sensitivity to variations in the pH and temperature, which can result in modifications in the color tone or even a loss of the pigment. Therefore, understanding this sensitivity to different conditions is crucial. Following the dyeing process, the textile is subjected to a pigment fixation procedure facilitated by a mordant, a chemical used to secure the pigment to the fiber before washing. Common mordants include the salts of aluminum, iron, and copper. However, the mordants may induce variations in the pigment shade.

Silk materials are particularly prone to microbial proliferation due to their inadequate antibacterial properties. Thus, the application of a pigment possessing antimicrobial activity is needed to enhance the fiber’s characteristics. Several studies have focused on the exploration of fiber dyeing utilizing pigments derived from *Streptomyces*. For instance, a notable study employed the pigment from marine-derived *Streptomyces cyaneofuscatus* in the dyeing of silk. The resultant investigation revealed that the treated silk not only retained its color but also maintained an impressive antibacterial efficacy of over 90% even after 20 washing cycles [20]. The melanin pigment derived from *Streptomyces glaucescens* was utilized in the dyeing of cotton fabric. The resultant dyed cotton exhibited a strong antioxidant activity, potentially leading to antimicrobial properties [33].

Hence, the aim of this study was to determine whether *Streptomyces* containing antibacterial pigments, isolated from soil under five tropical plant species, could provide a source of active natural dye for the silk fabric industry. To achieve this goal, actinobacteria were isolated and screened for pigment production on broken rice-solid-state cultivation. Bioassay-guided fractionation was employed to identify pigments with antibacterial properties. Then, the cytotoxicity and antibacterial activity on dyed silk fabric were determined. The outcome of this study will facilitate further research into the development of actinomycin-dyed silk fabrics.

## 2. Results

### 2.1. Isolation of Actinobacteria

In this study, soils collected at Romklao Botanical Garden under the Royal Initiative, Phitsanulok, Thailand, were used to isolate antibacterial pigment-producing actinobacteria. A total of 194 isolates of actinobacteria were obtained from under *Phanera siamensis* (18 isolates), *Cinnamomum parthenoxylon* (26 isolates), *Grevillea pteridifolia* (23 isolates), *Magnolia baillonii* (54 isolates), and *Magnolia rajaniana* (73 isolates). All isolates were then subjected to pigment-producing and antibacterial activity screening.

### 2.2. Screening for Pigment Production and Antibacterial Activity

**Figure 1:**
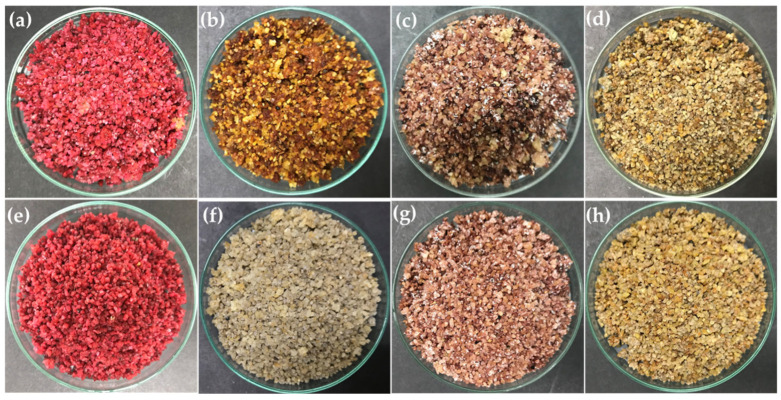
Pigment-producing *Streptomyces* cultivated on broken rice for seven days at room temperature, and then hot-air-dried at 60 °C for 3 h. (**a**) TBRC 15924, (**b**) TBRC 15925, (**c**) TBRC 15926, (**d**) TBRC 15927, (**e**) TBRC 15928, (**f**) TBRC 15929, (**g**) TBRC 15930, and (**h**) TBRC 15931.

In this work, 44 isolates out of 194 isolates were able to produce pigments when grown on broken rice as a solid substrate for seven days at ambient temperature (30 ± 2 °C). Among the forty-four isolates, eight crude extracts showed significant antibacterial activity against *Staphylococcus aureus* DMST 4745 and *S. epidermidis* TISTR 518. The pigment colors observed were pink to red (TBRC 15924, TBRC 15928), shades of brown (TBRC 15925, TBRC 15927, and TBRC 15931), gray (TBRC 15929), and purple (TBRC 15926, TBRC 15930) (Figure 1). The pigmented *Streptomyces* species were isolated from soil under *Magnolia baillonii* (TBRC 15924, TBRC 15927, TBRC 15931), *Magnolia rajaniana* (TBRC 15925, TBRC 15926, TBRC 15928, TBRC 15930), and *Cinnamomum parthenoxylon* (TBRC 15929).

Preliminary screening was undertaken on the ethyl acetate extracts of the 44 pigmented isolates. Eight isolates were then selected to obtain a more diverse profile and to explore compatible solvents for the extraction of bacterial pigments. In this experiment, eight extracts inhibited the growth of *Staphylococcus aureus* DMST 4745 and *S. epidermidis* TISTR 518, but not the Gram-negative bacteria *Escherichia coli* TISTR 527 or *Pseudomonas aeruginosa* DMST 15501. As a result of the antibacterial activity (Table 1), it was concluded that ethyl acetate is a suitable solvent for extracting antibacterial compounds. Crude extracts of TBRC 15925 alone demonstrated efficacy after extraction with ethyl acetate, although only extracts of TBRC 15924 extracted with 95% (*v*/*v*) ethanol inhibited the growth of *S. epidermidis* TISTR 518. Furthermore, the ethyl acetate crude extracts of TBRC 15927 and TBRC 15925 had the strongest antibacterial activity against *S. epidermidis*, with inhibition zones of 17.63 ± 1.48 mm and 16.50 ± 1.45 mm, respectively.

**Figure 2:**
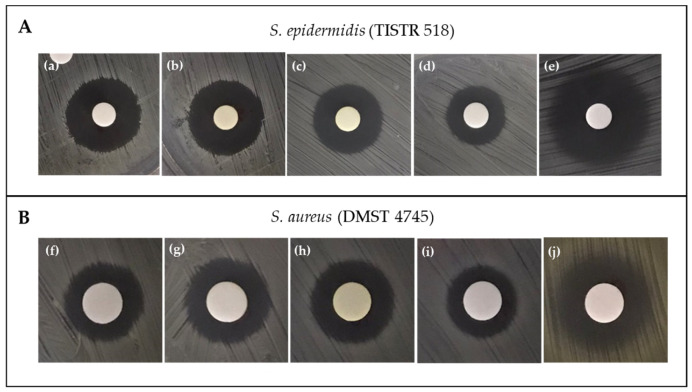
Growth inhibition zones of *Staphylococcus epidermidis* (TISTR 518) (**A**) and *S. aureus* (DMST 4745) (**B**) via the paper-disc-diffusion assay for crude extracts of TBRC 15927 using ethyl acetate soluble extract (**a**,**f**), methanol soluble extract (**b**,**g**), 95% ethanol extract (**c**,**h**), 70% ethanol (**d**,**i**), and chloramphenicol (**e**,**j**).

The bioassay tests performed on *S. aureus* DMST 4745 were similar to those performed on *S. epidermidis* TISTR 518. Hence, TBRC 15927 was chosen for further study based on the obtained weight of the crude extract and antibacterial properties of various solvent soluble extracts (Figure 2).

### 2.3. Identification of Actinobacteria and Phylogenetic Analysis

In this study, eight pigment-producing isolates capable of inhibiting selected pathogenic bacteria were used for molecular characterization. The 16S rRNA gene region sequencing data confirmed that these eight actinobacteria are members of the genus Streptomyces with high similarity (99.19–100%) (Table 2). They were identified as *Streptomyces adustus* TBRC 15929, *Streptomyces gramineus* TBRC 15927, *Streptomyces gramineus* TBRC 15931, *Streptomyces shenzhenensis* TBRC 15925, *Streptomyces* sp. TBRC 15924, *Streptomyces* sp. TBRC 15926, *Streptomyces* sp. TBRC 15928, and *Streptomyces* sp. TBRC 15930 (Table 2). 

**Figure 3:**
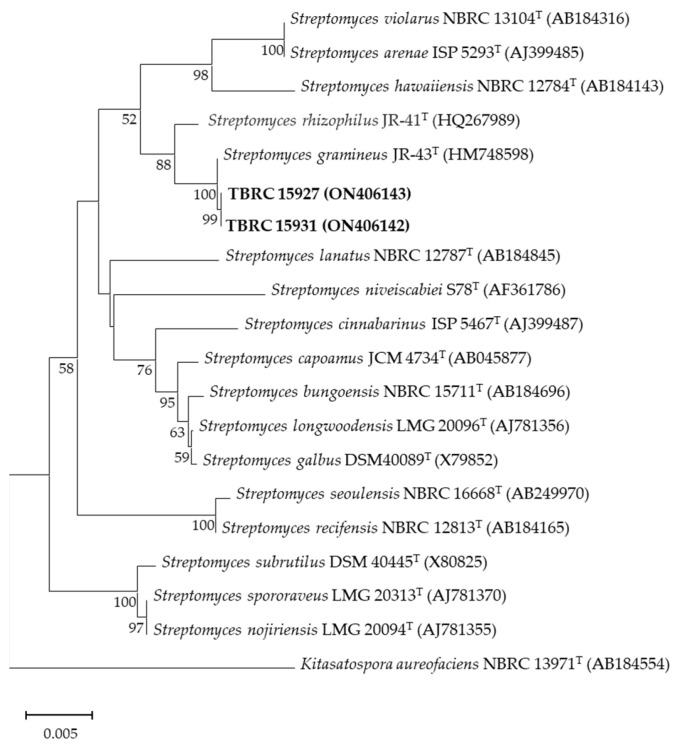
Phylogenetic tree using neighbor-joining approach based on 16S rRNA gene sequences of *Streptomyces* isolates. *Kitasatospora aureofaciens* NBRC 13971^T^ was used as an outgroup. Bootstrap values greater than 50% are displayed at branch nodes, based on 1000 replicates, and the scale bar indicates 0.005 nucleotide substitutions per site.

Phylogenetic analysis based on the 16S rRNA gene sequence comparisons revealed that TBRC 15927 and TBRC 15931 grouped within the genus *Streptomyces* where they showed a high similarity to *Streptomyces gramineus* JR-43^T^ (Figure 3).

### 2.4. Separation and Bioassay-Guided Fractionation 

#### 2.4.1. Paper-Disc-Diffusion Assay

Based on the antibacterial activity against *S. epidermidis* TISTR 518 and *S. aureus* DMST 4745, the ethyl acetate crude extract of TBRC 15927 was selected for fractionation. Separation was performed by column chromatography using silica gel as a stationary phase, and 26 fractions were eluted with methanol in a dichloromethane gradient (Table S2). Fraction 8 (red residue, 500 mg) was loaded onto a second silica gel column and 37 fractions were eluted with acetone in a chloroform gradient (Table S3). Fractions 10, 22–31, and 36 were further selected for paper-disc-diffusion assay based on the yield of the crude extract and chromatographic profile on thin layer chromatography. Fractions 22–31 (at concentration 5 µg/disc) impeded the growth of *S. epidermidis* TISTR 518 at 24–48 h. Fraction 28 (16.2 mg) had the biggest zone of inhibition of 20.70 ± 0.40 mm, followed by fractions 27 (47.8 mg), 26 (31.9 mg), and 23 (5.7 mg) (Table 3).

#### 2.4.2. TLC-Bioautographic Assay 

**Figure 4:**
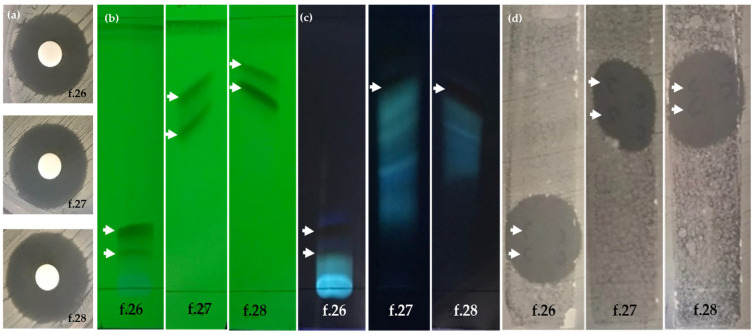
Isolation of metabolites from *Streptomyces gramineus* strain TBRC 15927 using thin-layer chromatography (TLC) of three fractions obtained by silica gel column chromatography. Solvent systems (% *v*/*v*) for TLC development; f.26: mobile phase: 3% methanol in dichloromethane, f.27: mobile phase: 5% methanol in dichloromethane, f.28: mobile phase: 7% methanol in dichloromethane (**a**) inhibition zone of fractions 26–28, (**b**) TLC plate under ultraviolet light at 254 nm, (**c**) TLC plate under ultraviolet light at 365 nm. Arrows (**b**,**c**) show active compounds that were developed on the TLC plate, and (**d**) bioautographic assay of three derived fractions (20 µg/band), arrow indicates the zone of inhibition of the antibacterial compound on the TLC plate.

Based on the growth inhibition in the disc-diffusion assay, the yield of the crude extract, and the chromatographic profile, fractions 10, 22–31, and 36 were investigated in a TLC-bioautographic assay. The result of the TLC-bioautographic assay was similar to the results from the disc-diffusion assay. However, the TLC-bioautographic assay revealed that antibacterial substances were located in fractions 22–31 (Figure 4). Among the 10 active fractions, fractions 26–28 were selected for the identification of active substances by LC-MS/MS. 

### 2.5. Detection of Actinomycins from Streptomyces gramineus TBRC 15927

The full-scan mass spectra of fractions 26–28 gave a high abundance of protonated molecules in the positive ion mode, and therefore these fractions were used for further studies that revealed three antibacterial compounds from fractions containing orange pigments. The compounds are detailed below.

**Figure 5:**
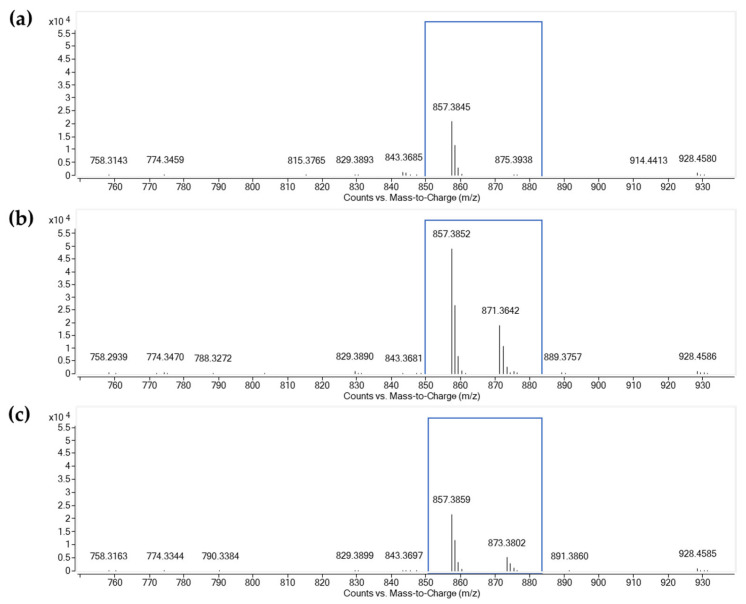
QTOF MS/MS spectra of actinomycins at fragmentor voltage of CID@40.0. The blue box highlights the targeted product ions specific to each identified actinomycin. (**a**) compound I was identified as actinomycin D, (**b**) compound II was identified as actinomycin X_2,_ and (**c**) compound III was identified as actinomycin X_0β_.

Compound I: the first component had a typical ESI-MS/MS of the compound with an intense ion at *m*/*z* 1255.6372 [M + H]^+^, which was identical to the results observed with actinomycin D (actinomycin X_1_). The [M + H]^+^ ion was selected as the precursor ion to obtain the QTOF MS/MS spectra. The loss of 28 Da was attributed to the elimination of a CO unit ([M + H–CO]^+^). The successive losses of 202, 97, and 99 Da gave the product ions at *m*/*z* 1053.5019, 956.4521, and 857.3845 (Figure 5a), indicating the occurrence of a Val-Pro-Sar-MeVal chain (398 Da) due to the successive losses of Sar-MeVal, Pro, and Val from the precursor ion. The product ions at *m*/*z* 657.2666 and 558.1992 indicated the occurrence of another Pro-Sar-MeVal residue, which was fragmented from the ions at *m*/*z* 956.4521 and 857.3845, respectively. The loss of another Val-Pro-Sar-MeVal chain (398 Da) from the ion at *m*/*z* 857.3845 generated the product ion at *m*/*z* 459.1300 which represented the mother nucleus structure. Other ions at *m*/*z* 399.2605, 300.1922, 203.1365, and 100.0390 were also detected, corresponding to the amino acid chain fragment ions of [(H-Val-Pro-Sar-MeVal-OH) + H]^+^, [(H-Pro-Sar-MeVal-OH) + H]^+^, and [(H-MeVal-Sar-OH) + H]^+^, respectively. Compound I was found in fractions 26–27.

Compound II: the results showed an accurate mass [M + H]^+^ ion at *m*/*z* 1269.6193 and gave the molecular formula C_62_H_84_N_12_O_17_. The molecular weight of compound II was 14 Da higher than that of compound I. The typical ions at *m*/*z* 956.4537, 857.3852, 657.2669, 399.2607, and 300.1921 were similar to those observed for compound I, corresponding to the loss of the Val-Pro-Sar-MeVal chain (398 Da). The presence of an ion at *m*/*z* 459.1307 indicated that the structure of compound II had the same mother nucleus as compound I. It was noted that an ion at *m*/*z* 871.3642 (Figure 5b) was detected from compound II but was not found in the spectra of compound I. The loss of 398 Da, producing the ion at *m*/*z* 871.3542 which was 14 Da higher than the ion at *m*/*z* 857.3852 for compound I, suggested that the second amino acid chain on the structure of compound II had a composition other than the Val-Pro-Sar-MeVal chain. Compound II was found in fractions 26–27.

Compound III: showed an accurate mass [M + H]^+^ ion at *m*/*z* 1271.6355 and gave the molecular formula C_62_H_86_N_12_O_17_. For this compound, the similar product ions at *m*/*z* 956.4543, 857.3859, 657.2684, 399.2610, 300.1923, and 459.1310 were consistent with the identification of the Val-Pro-Sar-MeVal; the loss of 398 Da gave the ion at *m*/*z* 873.3802 (Figure 5c) for compound III and 857.3815 for compound I, differing by 16 Da due to the different amino acid chain. Compound III was found in fractions 27–28.

In order to characterize the different amino acid chains, techniques based on in-source CID and QTOF MS/MS were applied for further investigations. Product ions at *m*/*z* 871.3642 and 873.3802 for compound II and compound III were particularly studied. By increasing the fragmentor voltage from 10 to 40 V, the ion at *m*/*z* 1269.6193 was fragmented and the fragment ion at *m*/*z* 871.3642 Da was produced. Then, the ion at *m*/*z* 871.3642 was selected as the precursor ion and an MS/MS spectrum was generated (Figure 5b). The losses of 97, 117, 99, and 99 Da, producing ions at *m*/*z* 774.3470, 657.2669, 558.1992, and 459.1307, represented the successive losses of Pro, L-Val, Val, and Val from the ion at *m*/*z* 871.3642, respectively. Therefore, the amino acid chain was assigned as Val-Val-L-Val-Pro. Due to the unavailability of a reference compound, compound II was tentatively identified as actinomycin X_2_. We employed the same method and obtained the MS/MS spectrum of the ion at *m*/*z* 873.3802. The fragment ions at *m*/*z* 774.3470, 657.2669, 558.1992, and 459.1310 corresponded to the fragments of compound III due to the losses of 99, 117, 99, and 99 Da, indicating the presence of the amino acid chain Val-Val-L-Val-Val chain and mother nucleus structure. Therefore, compound III was tentatively assigned as actinomycin X_0β_. The accurate mass of [M + H]^+^ and product ions of the actinomycins are presented in Table 4.

### 2.6. Color Characteristics 

**Figure 6:**
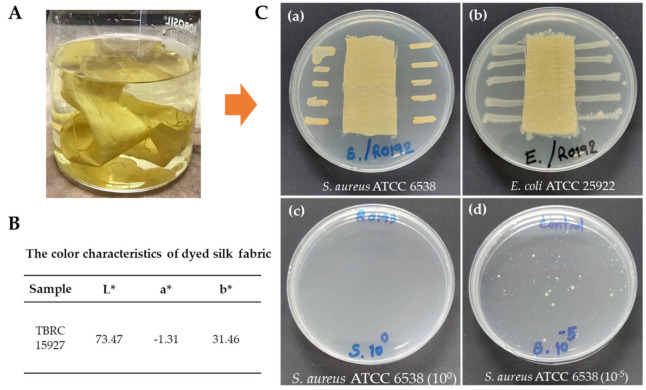
Properties of dyed silk fabric. (**A**) Silk fabric after dyeing, (**B**) table presenting the color characteristics of dye silk fabric [color strength, L*: lightness, a*: (+ value = red, − value = green) b*: (+ value = yellow, − value = blue)]. (**C**) Antibacterial activity of coated silk fabric using qualitative (AATCC 147-2011) (**a**,**b**) and quantitative (AATCC 100-2019) test methods (**c**,**d**) and 24 h incubation.

Upon completion of the dyeing process, the dyed silk had a yellowish appearance (Figure 6A). The colorimetric data of the dyed silk by the crude ethyl acetate extract of TBRC 15927 were performed with multiple measurements (n = 3). As illustrated in Figure 6B, the CIE Lab value showed high L* values, indicating that the dyed silk fabric exhibited a notable level of brightness. Furthermore, the a* and b* values for silk fabrics dyed with TBRC 15927 demonstrated a shift toward the green coordinate within the green–yellow zone of the CIE Lab color space. 

### 2.7. Antibacterial Activity of Dyed Silk Fabric

The results of the qualitative test method (AATCC 147-2011) demonstrated that the silk fabric dyed with the TBRC 15927 crude extract was active against *S. aureus* ATCC 6538 but not *E. coli* ATCC 25922 (Figure 6C(a,b)). When laid over bacteria colonies on agar, the dyed fabric inhibited the growth of *S. aureus* ATCC 6538, forming an inhibition zone of 7.4 mm. From the quantitative (AATCC 100-2019) tests, *S. aureus* ATCC 6538 had a percentage reduction value of 99.99%, while for *E. coli* ATCC 25922, the reduction value was 47.90%. The number of bacteria in the untreated fabric control were not reduced (Figure 6C(c,d)).

### 2.8. Cytotoxicity Assay

The ethyl acetate crude extract of TBRC15927 and doxorubicin cytotoxicity were analyzed via the MTT assay using the mouse fibroblast L-929 cell line. Actinomycins mixed with unknown compounds obtained from the crude extract significantly decreased the cell viability in a concentration-dependent manner, showing in vitro cytotoxic activity against the mouse fibroblast L-929 cell line with the IC_50_ value of 0.029 ± 0.008 µg/mL. The crude extract displayed a high cytotoxicity compared to doxorubicin at a concentration of 0.402 ± 0.040 µg/mL (Table 5).

## 3. Discussion

Due to their capacity to produce biologically active secondary metabolites, actinobacterial communities are one of the prokaryote sources that have received particular attention. Several actinobacterial groups are stable in bulk soil and plant rhizospheres, but *Streptomyces* spp. are the most prevalent because of their potential to produce antibiotics. In this study, we isolated 194 actinobacterial strains from soil under perennial plants growing in a botanical garden in Thailand. We found that starch casein agar was the best medium for isolating actinobacteria (81 isolates: 41.75%), which was similar to the studies of Zothanpuia [34] and Geetanjali & Jain [35]. Starch casein agar is most commonly employed for isolating saccharolytic bacteria because it contains a variety of nutrients, vitamins, and sea salt that support the growth of terrestrial [36,37] and marine microorganisms [38]. Among the soils sampled, *Streptomyces* isolated from under *Magnolia baillonii* and *M. rajaniana* harbored pigment producers with antibacterial properties (Table S1). In our study, TBRC 15927 and TBRC 15931, which were isolated from under *Magnolia baillonii*, exhibited pigment-producing antibacterial properties. These findings suggest that different root environments may favor particular assemblages of *Streptomyces*.

In previous studies on the production of secondary metabolites, many natural substrates have been utilized due to their numerous advantages, such as producing more stable products, requiring less energy, and facilitating downstream processing [39]. In our screening program, among 194 isolates, 44 pigment-producing isolates were cultured on broken rice as a natural solid substrate. The examination of forty-four crude ethyl acetate extracts against Gram-positive and Gram-negative bacteria resulted in the selection of eight isolates for sequential maceration extract. These isolates tested positive for antibacterial activity against Gram-positive bacteria and they were identified as *Streptomyces*. Two of these isolates, *Streptomyces* sp. TBRC 15927 and TBRC 15931, contained a similar group of actinomycin called actinomycin X_2_ (Figure S1).

According to a phylogenetic analysis of the 16S RNA gene region, isolates TBRC 15927 and TBRC 15931 were grouped in the same clade and exhibited 100% homology to *Streptomyces gramineus* strain JR-43^T^. Strain JR-43^T^ was initially described as a novel species isolated from the rhizosphere soil of bamboo (*Sasa borealis*) [40]. This strain was reported to produce a yellow pigment on International *Streptomyces* Project (ISP)-4 and was able to support the growth of phytopathogenic *Xanthomonas* spp. However, the antibacterial agent remains unknown. Pigment production on solid media was regularly observed when actinobacteria from different resources were isolated. There have been reports of various colors, including blue, yellow, and red [40,41,42].

Silk fabric is a popular and widely used material in many countries. However, it is important to note that bacteria can inhabit this cloth due to its natural fibers. Natural fiber-based cloths can play a significant role in transmitting pathogens [43]. According to research, bacteria can survive on 100% cotton, blended cotton, and silk fabrics for various lengths of time due to the fabric structures [44]. Additionally, fabrics that absorb liquid more effectively also provide a more favorable environment for bacterial growth. Although plants are the primary source of natural dye, their availability is limited. As they are renewable and biodegradable, microbial dyes can be substituted. Microbial dyes may possess antimicrobial properties, so they could be a good and readily accessible source of natural dyes [45].

Only a few of the many research efforts seeking pigment-producing isolates with antimicrobial activity have had a focus on their end use as fabric dyes [46]. It is, therefore, highly desirable to limit the growth of bacterial contamination while fabrics are being used and stored. This study successfully selected the best strain using dereplication techniques such as rapid screening on broken rice as a solid substrate culture, bioassay-guided fractionation, and MS analysis to find the active substances in crude pigments. We conclude that broken rice is a cost-effective production method for antibacterial production. Solid substrates play a significant role in the production of various antibiotics, including actinomycins [16,47,48]. Our research has effectively isolated an antibacterial pigment from *Streptomyces gramineus* TBRC 15927 that gives raw silk a light-greenish yellow sheen.

In our study, the actinomycin X complex was present in the ethyl acetate crude extracts. Actinomycins, a family of chromogenic lactone peptide antibiotics that differ only in the peptide portion of the molecule, were first reported in 1940 [25]. More than 40 actinomycins have been widely researched and clinically applied due to their excellent antibacterial, antitumor, and antiviral activities, but few have been used as textile colorants [49]. In this study, actinomycin D, actinomycin X_2,_ and actinomycin X_0β_ were identified by a comparison of their MS data with those reported in the literature. Similarly, actinomycin D, actinomycin X_2,_ and actinomycin X_0β_ were identified by MS and NMR techniques from *Streptomyces heliomycini* [50]. In our study, we employed TLC-bioautography, a method that is not only simple and inexpensive but also highly sensitive and specific [51]. This technique enabled the identification of actinomycin D and X_0β_ from *Streptomyces* sp. Av-R5, as well as actinomycin D and X_2_ from *Streptomyces smyrnaeus* UKAQ_23 [19,52]. Due to the presence of actinomycins (actinomycin D, X_2,_ and X_0β_) in the ethyl acetate crude extract of TBRC 15927, the antibacterial activity against *Staphylococcus aureus* DMST 4745 and *Staphylococcus epidermidis* TISTR 518 was highly effective. Actinomycin D has a bacteriostatic effect on many Gram-positive bacteria [53] and has been shown to inhibit biofilm formation in *S. aureus* [54] and *S. epidermidis* [55]. Actinomycin X_2_ and X_0β_ have often been isolated along with actinomycin D [31,50,52] and recently, actinomycin L, a new member of the actinomycin family, was reported from *Streptomyces* sp. MBT27 [31]. In our study, actinomycin-dyed silk fabric exhibited antibacterial activity greater than 99.99% against *S. aureus* ATCC 6538, as measured by the AATCC 100-2019 quantitative test method. Chen et al. found that silk fabric dyed with actinomycin X_2_ showed a high antibacterial activity (>95%) against *S. aureus* when tested using AATCC 100-2012 [20].

Our results indicate that the crude extract from *Streptomyces gramineus* TBRC 15927 was not toxic to the L-929 mouse fibroblast cells, with an IC_50_ value of 0.029 ± 0.008 µg/mL (0.029 ppm). According to the report by Ramirez-Rodriguez et al. [56], the toxicity of the L-929 mouse fibroblast cells was tested with Doxorubicin (1–25 ppm) as a standard substance. They concluded that crude extracts with an IC_50_ value of less than 50 ppm are not significantly toxic to mammal cells. Several yellow microbial pigments, such as arugosin A [46], carotenoids [57], flavins [58], and melanin [46], have potential as textile dyes. Predominantly classified as secondary metabolites, these pigments may possess inherent biological activities, including some antimicrobial properties. Furthermore, their photostability and thermal stability qualify them as suitable candidates for textile colorants. Despite the potential, the successful incorporation of these pigments into textile matrices remains challenging. At present, microbial pigments of red, pink, violet, blue, and brown spectra have been effectively applied to textiles. Moreover, it is noteworthy that the majority of microbial pigments necessitate the use of mordants to enhance their substantive properties on textiles.

In our investigations, however, we have identified that the crude ethyl acetate extract derived from TBRC 15927 exhibits the capacity to dye silk without the requirement of any additional mordanting agents. The present study confirms the presence of actinomycin D, actinomycin X_2_, and actinomycin X_0β_ in the extracts, which are likely contributors to the observed pigmentation. However, the complexity of natural product extracts raises the possibility of other pigmented molecules. To better understand their specific contributions, the researchers propose further purification and testing of individual color properties and combinations. Further studies will be conducted to isolate and identify pigmented molecules, enhancing the research.

This finding potentially initiates a new trajectory in the exploration of mordant-free microbial pigments for silk textile applications. Further studies are needed to validate these promising initial results and to explore the full potential of these naturally derived microbial colorants.

## 4. Materials and Methods

### 4.1. Soil Sampling and Isolation of Actinobacteria

Soil samples were collected from under *Phanera siamensis* (K.Larsen & S.S.Larsen) Mackinder & R. Clark, *Cinnamomum parthenoxylon* (Jack) Meisn., *Grevillea pteridifolia* Knight, *Magnolia baillonii* Pierre, and *Magnolia rajaniana* (Craib) Figlar growing in the Romklao Botanical Garden under the Royal Initiative, Phitsanulok, Thailand. To avoid contamination with other surfaces, soil samples (100 g) were taken in triplicate at 10–20 cm depth from under the canopy of each plant. Each soil sample was allowed to air-dry, then the soil was sieved through a 0.2 mm sieve and stored at 4 °C. Ten-fold serial dilutions of the soil samples were conducted using sterile distilled water. Soil suspensions (100 µL) from appropriate dilutions (10^−1^–10^−4^) were spread onto actinomycete isolation agar (AIA, composition per liter: sodium propionate: 4.0 g, sodium caseinate: 2.0 g, K_2_HPO_4_: 0.5 g, L-asparagine: 0.1 g, MgSO_4_·7H_2_O: 0.1 g: FeSO_4_·7H_2_O: 0.001 g, agar: 15 g; pH 7.0), oatmeal agar (OMA, composition per liter: oatmeal: 30 g, agar: 15 g; pH 7.2), peatmoss extract agar (composition per liter: 200 g of peatmoss was extracted by 50 mM NaOH solution and agar 15 g; pH 5.0), and starch casein agar (SCA, composition per liter: soluble starch: 10 g, casein hydrolysate: 0.3 g, KNO_3_: 2.0 g, NaCl: 2.0 g, K_2_HPO_4_: 2.0 g, MgSO_4_.7H_2_O: 0.05 g, CaCO_3_: 0.02 g, FeSO_4_.7H_2_O: 0.01 g, agar: 15 g; pH 7.2). To selectively isolate actinobacteria, media were supplemented with 50 µg/mL cycloheximide and 100 µg/mL nalidixic acid [59,60]. The plates were incubated at 30 ± 2 °C until pure single colonies appeared (7–14 days). All colonies obtained from the four different media were streaked on International *Streptomyces* Project-2 (ISP-2) agar (composition per liter: malt extract: 10 g, yeast extract: 4 g, dextrose anhydrous: 4 g, agar: 15 g; pH 7.2), to compare the colonies using a stereomicroscope. Based on pigmentation on ISP-2 agar and colony form, 194 isolates were selected for preliminary study. Where more than one colony from a single source was identical in appearance, we chose one. Single colonies with filamentous features were selected and streaked on ISP-2 agar and incubated at 30 ± 2 °C for 7 days. The actinobacterial isolates were preserved at −80 °C in the presence of glycerol (25% *v*/*v*) and maintained in the Thailand Bioresource Research Center (TBRC), Thailand.

### 4.2. Screening for Pigment Production and Antibacterial Activity of Pigments

#### 4.2.1. Solid-State Fermentation

As a substrate for solid-state fermentation, broken rice purchased locally was used to perform preliminary screening for pigment producing isolates. The broken rice was thoroughly washed with distilled water and 30 g of imbibed broken rice was added to glass bottles. The substrate was autoclaved for 20 min at 121 °C. Three actinobacterial mycelial plugs (5 mm diameter) from 3-day-old fresh cultures on ISP-2 agar were inoculated into each bottle and incubated at 30 ± 2 °C for 7 days. The solid culture was air-dried in a hot-air oven at 60 °C for 3 h and then ground. 

#### 4.2.2. Production of Ethyl Acetate Crude Extract

The dried samples obtained from Section 4.2.1 were extracted using a maceration method as described in previous studies [61]. The samples were submerged in ethyl acetate in a static condition for 8–12 h and stored in the dark. After filtering each sample, the filtrate was evaporated using a rotary evaporator to produce the ethyl acetate crude extract. The obtained crude extract was then uniformly adjusted to a concentration of 5 mg/mL using ethyl acetate. 

#### 4.2.3. Preparation of Media

Nutrient agar was prepared according to the manufacturer’s instructions (HIMEDIA) using aseptic technique. Before use, sterilized nutrient agar was poured into Petri dishes and allowed to solidify at room temperature. Prepared agar plates were stored in a cool room until use.

#### 4.2.4. Test Microorganisms

*Escherichia coli* TISTR 527 and *Staphylococcus epidermidis* TISTR 518 were obtained from the Thailand Institute of Scientific and Technological Research (TISTR).

*Pseudomonas aeruginosa* DMST 15501 and *Staphylococcus aureus* DMST 4745 were obtained from the Department of Medical Sciences Thailand (DMST).

#### 4.2.5. Bioassay for Antibacterial Activity

Antibacterial activity of the extracted pigment was examined by Kirby–Bauer disc diffusion susceptibility test and paper-disc-diffusion assay as described in [62,63]. The inoculum suspension was prepared by selecting several colonies on the surface of the solid nutrient agar and transferring three loopfuls into 5 mL of sterilized nutrient broth in 18 × 150 mm test tubes. The cultures were incubated at 37 °C at 150 rpm on an incubator shaker for 24 h. The bacterial suspension was diluted with sterilized nutrient broth to an OD_600_ of 0.1, which corresponds to approximately 10^5^–10^8^ CFU/mL. A total of 100 µL of bacterial suspensions was spread on agar plates. 

Then, 20 µL of the ethyl acetate crude extracts (100 µg/disc) obtained from Section 4.2.2 was loaded onto sterile filter paper discs (6 mm), and the discs were air-dried in a biosafety cabinet for 30 min. Paper discs containing crude extracts were carefully placed on the bioassay plates. The bioassay plates were kept in an incubator chamber at 37 ± 2 °C for 24 h. Later, the diameter of the inhibition zone was measured (in mm) using a Vernier caliper. Chloramphenicol was used as a positive control (30 µg/disc). Experimental data for antibacterial activity (inhibition zone size) were compared with the control using Duncan’s new multiple range test (*p* < 0.05, n = 3). The most potent pigment-producing strains with antibacterial activity and yield of the crude extract were selected for further study.

### 4.3. Identification of Actinobacteria and Phylogenetic Analysis

In this study, eight isolates of *Streptomyces* with ability to produce pigments and with antibacterial activity (results obtained from Section 4.2.1 and Section 4.2.5) were selected for identification. Genomic DNA was extracted and used for amplifying the 16S rRNA gene with the 27F primer (5′-AGA GTT TGA TCM TGG CTC AG-3′) and 1492R primer (5′-TAC GGY TAC CTT GTT ACG ACT T-3′) [64], and the purified PCR products were commercially sequenced at Macrogen, Korea. The 16S rRNA gene sequences obtained were compared to phylogenetic neighbors in the EzBioCloud database (http://www.ezbiocloud.net/ accessed on 7 July 2022). Phylogenetic trees based on a neighbor-joining approach were constructed and analyzed using the MEGA software package (Version 11) [65]. Bootstrap analysis with 1000 resampled datasets was used to evaluate the resultant tree topology. The nucleotide sequences were then submitted to GenBank database to obtain accession numbers.

### 4.4. Pigment Production and Extraction 

The findings obtained from Section 4.2.5 were the basis of this experiment. Eight strains of actinobacteria obtained from glycerol stock (25% *v*/*v*) were streaked on International *Streptomyces* Project ISP-2 agar and incubated at 30 ± 2 °C for three days. Later, each isolate was inoculated into ISP-2 broth and incubated for 3 days at 30 ± 2 °C on an orbital shaker at 150 rpm for use as seed culture. A 10% (*v*/*w*) inoculum was introduced to 1 L Erlenmeyer flasks containing 250 g of sterilized broken rice and incubated for 7 days at 30 ± 2 °C. After the incubation period, the solid culture on the broken rice was placed in a hot-air incubator at 60 °C for 3 h. Each sample was sequentially macerated and successively extracted with five organic solvents: ethyl acetate, methanol, 70% (*v*/*v*) ethanol, and 95% (*v*/*v*) ethanol in increasing polarity order [66,67]. The ratio of the dried sample and solvent was 1:2 (*w*/*v*). The extracts were left overnight at room temperature. In order to obtain the crude extracts, the samples were filtered and evaporated in a rotary evaporator and stored at 4 °C.

### 4.5. Separation

Ethyl acetate crude extract (3 g) was separated by column chromatography (CCI; 33.5 × 3 cm) on silica gel with methanol in a dichloromethane gradient and collected every 10 min to obtain 26 fractions (Table S2). Separation of fraction 8 (500 mg) was conducted using a silica gel column (CCII; 23.5 × 1.5 cm) and eluted with acetone in a chloroform gradient. Fractions were collected every 2 min to obtain 37 fractions (Table S3). All fractions were subsequently evaluated using paper-disc-diffusion and bioautographic assay [17,68].

### 4.6. Bioassay-Guided Fractionation 

#### 4.6.1. Paper-Disc-Diffusion Method

In this study, *Staphylococcus epidermidis* TISTR 518 was selected to test for antibacterial activity with samples from 37 fractions using the paper-disc diffusion method as described earlier. These samples obtained from CCII were prepared at 5 µg/disc. A disc containing 20 µL of solvents was used as a negative control, while streptomycin (5 µg/disc) and chloramphenicol (5 µg/disc) were used as positive controls.

#### 4.6.2. TLC-Bioautographic Assay

Based on its susceptibility, *Staphylococcus epidermidis* TISTR 518 was selected as the test organism in this study. To prepare the bioassay plates, three loopfuls from the colonies from nutrient agar were transferred into 5 mL of sterilized nutrient broth in an 18 × 150 mm test tube. The culture was incubated at 37 °C at 150 rpm on an incubator shaker for 24 h. The bacterial suspension was diluted with sterilized nutrient broth to an OD_600_ of 0.1, which corresponds to approximately 10^7^ CFU/mL. Bacterial density was adjusted using sterilized nutrient broth. The culture was inoculated on the surface of the nutrient agar plates.

Following Yamaç and Bilgili [69], the TLC-bioautographic test was used in this study. Based on the findings from the paper-disc-diffusion method and the dried weight of the eluted fractions, the selected fractions (fractions 10, 22–31, and 36) were subjected to TLC using aluminum foil-backed silica gel 60 F_254_ plates (Merck KGaAA^®^, Darmstadt, Germany, 2 × 9 cm). A sample from the selected fractions was dissolved in methanol:dichloromethane (1:1) to give 200 μg/mL of solution. Then, 10 μL of solution was applied to the plate which delivered a concentration of 20 μg/spot. Each fractional sample was developed on a mobile phase using an optimized system. Sample 10 was set on a TLC plate utilizing a mixture of chloroform and acetone (1:1) as the mobile phase. In contrast, samples 22–31 and 36 were subjected to varying concentrations of methanol in dichloromethane (Table S4). The TLC strips were then aseptically placed on the nutrient agar surface which was already seeded with the test bacterium. The plates were left at room temperature for 1 h to allow the active metabolites from the TLC strips to diffuse. Following that, the plates were incubated for 24 h at 37 °C while being observed for the growth of inhibitory zones. The molecular weight of compounds in the active fraction was obtained by mass spectrometry.

### 4.7. LC-ESI-Q-TOF-MS/MS 

From the results of the bioautographic assay, fractions 26–28 were selected for LC-MS/MS analysis on Q-TOF (Agilent Technologies, Palo Alto, CA, USA) as described by Jumpathong et al. [16]. Dereplication of those features was performed by comparing the accurate masses and fragmentation patterns obtained in MS/MS analysis against the chemistry database ChemSpider and by comparison with MS data previously reported in the literature [70]. 

### 4.8. Color Characteristics and Determination of Antibacterial Activity on Dyed Silk Fabric

#### 4.8.1. Color Characteristics

Silk thread was obtained from local villagers and used to weave undyed fabric. Silk fabric pieces (21 × 29.7 cm, 71.98 g) were boiled for 20 min to remove fat before drying. The fabric was soaked for 1 h at room temperature in a solution of the crude ethyl acetate extract obtained from TBRC 15927 (1.67 g) dissolved in 200 mL of ethyl acetate. After the samples were dyed, the fabrics were rinsed with distilled water and air-dried on the bench. Each test was performed twice. The dyed silk was assessed using a portable colorimeter CR400 (Konica Minolta, Japan) with CIELAB color system (Hunter Associates Laboratory Inc., Reston, VA, USA). The CIE Lab value is presented by L* (lightness), a* (with positive values corresponding to red and negative values to green), and b* (where positive values indicate yellow and negative values denote blue).

#### 4.8.2. Determination of Antibacterial Activity on Dyed Silk Fabric

The antibacterial activity of the bioactive crude extract of TBRC 15927 on the silk fabric was tested against *Staphylococcus aureus* ATCC 6538 and *Escherichia coli* ATCC 25922 using the standard methods of the American Association of Textile Chemists and Colorists (AATCC), namely the AATCC 147-2011 [71] qualitative test and the AATCC 100-2019 [72] quantitative test. These are called an agar diffusion test and suspension test, respectively. 

### 4.9. Cytotoxicity Assay

The standard colorimetric MTT (3-[4,5-dimethylthiazol-2-yl]-2,5-diphenyltetrazolium bromide) assay with the L-929 cell line (mouse fibroblast) was performed. Briefly, L-929 cells were grown in DMEM (Invitrogen) supplemented with 50 units/mL penicillin (Invitrogen) and 100 µg/mL streptomycin (Invitrogen) and kept at 37 °C and 5% CO_2_ in a humidified incubator. Cells were dispensed into 96-well plates at a density of 2 × 10^4^ cells/well. 

The ethyl acetate crude extract of *S. gramineus* TBRC 15927 was tested at concentrations of 0.008, 0.016, 0.032, 0.064, and 0.128 µg/mL. The cell viability was then determined using a cytotoxicity assay according to the procedure described by Sriwiriyajan et al. [73]. A microplate spectrophotometer was used to measure absorbance at 570 and 650 nm. Doxorubicin was used as the positive control. Cytotoxicity was expressed as the concentration of the compound inhibiting growth by 50% (IC_50_).

### 4.10. Statistical Analysis 

All tests were conducted in three replicates. Data were subjected to analysis of variance (ANOVA) followed by Duncan’s multiple range test (*p* < 0.05) using SPSS Statistics 17.0 for Windows (Trial version).

## 5. Conclusions

*Streptomyces gramineus* TBRC 15927 isolated from soil under *Magnolia baillonii* produced a yellow pigment with antibacterial properties. The active molecules identified in the crude ethyl acetate extract were actinomycin D (or X_1_), actinomycin X_2_, and actinomycin X_0β_. Pigments containing actinomycins as antibacterial agents have potential for commercial use as silk fabric dyes. This study provides a scientific basis for applying pigments derived from *Streptomyces* to silk fabric and identifies a role for the actinomycin X complex in the production of natural dyes for the textile industry.

## Figures and Tables

**Table 1:** Antibacterial activity against *Staphylococcus aureus* and *S. epidermidis* of crude pigments obtained from sequential extraction of eight *Streptomyces* isolates.

	Diameter of Inhibition Zone (mm) ^§^							
*Streptomyces* Crude Pigment	*S. epidermidis* (TISTR 518)				*S. aureus* (DMST 4745)			
	Ethyl Acetate	Methanol	95% Ethanol	70% Ethanol	Ethyl Acetate	Methanol	95% Ethanol	70% Ethanol
TBRC 15924	-	-	5.90 ± 0.44 ^d^	-	-	-	-	-
TBRC 15925	16.50 ± 1.45 ^bc^	-	-	-	11.00 ± 1.32 ^aA^	-	-	-
TBRC 15926	9.87 ± 0.32 ^deA^	7.30 ± 0.26 ^dC^	8.27 ± 0.25 ^cB^	6.83 ± 0.59 ^dC^	6.60 ± 0.36 ^cA^	-	-	-
TBRC 15927	17.63 ± 1.48 ^bAB^	18.07 ± 1.01 ^bA^	16.20 ± 0.35 ^bB^	12.37 ± 0.35 ^cC^	11.50 ± 0.87 ^aAB^	11.75 ± 0.68 ^aAB^	12.17 ± 0.76 ^aA^	10.35 ± 1.02 ^bB^
TBRC 15928	9.00 ± 0.30 ^ef^	-	-	-	6.30 ± 0.20 ^cA^	-	-	-
TBRC 15929	15.77 ± 0.21 ^cA^	15.27 ± 0.75 ^cA^	-	15.50 ± 0.48 ^bA^	11.73 ± 0.59 ^aA^	11.97 ± 0.95 ^aA^	5.70 ± 0.17 ^bB^	11.47 ± 0.15 ^aA^
TBRC 15930	8.27 ± 0.75 ^fA^	6.03 ± 0.21 ^dB^	8.03 ± 0.45 ^cA^	6.08 ± 0.41 ^dB^	6.50 ± 0.46 ^cA^	-	6.20 ± 0.53 ^bA^	-
TBRC 15931	10.77 ± 0.12 ^d^	-	-	-	8.57 ± 0.85 ^bA^	-	-	-
Control	21.27 ± 0.58 ^a^*	21.27 ± 0.58 ^a^	21.27 ± 0.58 ^a^	21.27 ± 0.58 ^a^	12.27 ± 0.81 ^a^	12.27 ± 0.81 ^a^	12.27 ± 0.81 ^a^	12.27 ± 0.81 ^a^

^§^ Values are presented as mean ± standard deviation. Control: chloramphenicol at a concentration of 30 µg/disc. -, no antibacterial activity. * Different lower-case letters in each column indicate significant differences from Duncan’s multiple range test (*p* < 0.05, n = 3). Different upper-case letters in each row indicate significant differences from Duncan’s multiple range test (*p* < 0.05, n = 3).

**Table 2:** The molecular characterization of actinobacteria isolated from soils by 16S rRNA gene sequencing.

Isolate Code	NCBI Accession Numbers	Amplified 16S rRNA Gene (bps)	Closely Related Taxa and NCBI Accession Number	Similarity (%)	Chosen Nomenclature
TBRC 15924	ON406138	1434	*Streptomyces cinnamoneus* NBRC 12852^T^	99.72	*Streptomyces* sp.
TBRC 15925	ON406139	1459	*Streptomyces shenzhenensis* 172115^T^	99.72	*S. shenzhenensis*
TBRC 15926	ON406140	1447	*Streptomyces aquilus* GGCR-6^T^	99.64	*Streptomyces* sp.
TBRC 15927	ON406141	1433	*Streptomyces gramineus* JR-43^T^	100.0	*S. gramineus*
TBRC 15928	ON406142	1366	*Streptomyces netropsis* NBRC 3723^T^	99.19	*Streptomyces* sp.
TBRC 15929	ON406143	1451	*Streptomyces adustus* WH-9^T^	99.93	*S. adustus*
TBRC 15930	ON406144	1436	*Streptomyces aquilus* GGCR-6^T^	99.64	*Streptomyces* sp.
TBRC 15931	ON406145	1471	*Streptomyces gramineus* JR-43^T^	100.0	*S. gramineus*

**Table 3:** Growth inhibition of *Staphylococcus epidermidis* (TISTR 518) from column chromatography fractions of *S. gramineus* TBRC 15927 using the paper-disc-diffusion method.

Sample	Diameter of Inhibition Zone (mm) ^¥^	
	24 h	48 h
Fraction 22	15.50 ± 0.78 ^e^	16.07 ± 0.12 ^f^
Fraction 23	17.37 ± 0.35 ^c^	18.07 ± 0.42 ^c^
Fraction 24	16.83 ± 0.12 ^cd^	17.07 ± 0.12 ^d^
Fraction 25	16.70 ± 0.10 ^d^	16.77 ± 0.59 ^de^
Fraction 26	18.23 ± 0.15 ^b^	18.33 ± 0.15 ^bc^
Fraction 27	18.67 ± 0.21 ^b^	18.80 ± 0.17 ^b^
Fraction 28	20.30 ± 0.30 ^a^	20.70 ± 0.40 ^a^
Fraction 29	16.43 ± 0.29 ^d^	16.40 ± 0.10 ^ef^
Fraction 30	15.50 ± 0.35 ^e^	15.37 ± 0.12 ^g^
Fraction 31	10.83 ± 0.29 ^g^	9.23 ± 0.06 ^i^
Chloramphenicol	10.43 ± 0.57 ^g^	-
Streptomycin	11.90 ± 0.30 ^f^*	10.50 ± 0.10 ^h^

^¥^ The value represents the mean ± standard deviation of three replicate determinations. Controls: chloramphenicol and streptomycin (5 µg/disc). -, no antibacterial activity. * Different lower-case letters in each column indicate significant differences from Duncan’s multiple range test (*p* < 0.05, n = 3).

**Table 4:** Accurate mass of [M + H]^+^ and product ions of the actinomycins analyzed by HPLC/ESI-QTOF MS/MS of actinomycin compounds.

Compound	*t*_R_ (min)	Measurement (*m/z*)	Calculated (*m/z*)	Diff (ppm)
Compound I: Actinomycin D (X_1_)				
Fraction 26	28.430	1255.6372	1255.6358	−1.15
Fraction 27	28.294	1255.6385	1255.6358	−2.19
Fraction 28	28.249	1255.6379	1255.6358	−1.71
Standard Actinomycin D (X_1_)	28.411	1255.6368	1255.6358	−0.84
Compound II: Actinomycin X_2_				
Fraction 26	28.590	1269.6193	1269.6150	−3.37
Fraction 27	28.306	1269.6218	1269.6150	−5.34
Fraction 28	28.357	1269.6177	1269.6150	−2.11
Standard Actinomycin X_2_	28.383	1269.6173	1269.6150	−1.80
Compound III: Actinomycin X_0β_				
Fraction 27	28.129	1271.6326	1271.6307	−1.52
Fraction 28	28.167	1271.6332	1271.6307	−1.99

**Table 5:** IC_50_ values, determined by MTT assay of ethyl acetate crude extract of TBRC 15927.

Sample	IC_50_ Values from MTT (µg/mL)
Crude extract TBRC15927	0.029 ± 0.008 *
Doxorubicin	0.402 ± 0.040

Data are shown as means (n = 3) ±SD from three independent experiments. * *p* value of <0.05 Doxorubicin: positive control.

## Data Availability

Not applicable.

## Supplementary Materials

The following supporting information can be downloaded at: https://www.mdpi.com/article/10.3390/molecules28165949/s1, Figure S1: QTOF MS/MS spectra of actinomycins at fragmentor voltage of CID@40.0, (A) actinomycin X_2_ detected in the crude ethyl acetate extract of TBRC 15931 (code TN166), (B) sample of standard actinomycin X_2_; Table S1: detail of *Streptomyces* isolated from soils and accession numbers in GenBank; Table S2: fractionation using silica gel column chromatography no. 1 column (size 33.5 × 3 cm 3000 mg); Table S3: fractionation using silica gel column chromatography no. 2 column (size 23.5 × 1.5 cm, 500 mg). Table S4: Solvent systems (% *v*/*v*) for dissolving the selected fractions and for TLC development.

## Sample Availability

Samples of the compounds are available from the authors.

## References

[1] Saxena S., Raja A.S.M. (2014). Natural dyes: Sources, chemistry, application and sustainability issues. Roadmap to Sustainable Textiles and Clothing: Eco-Friendly Raw Materials, Technologies, and Processing Methods.

[2] Mohammad Azmin S.N.H., Sulaiman N.S., Mat Nor M.S., Abdullah P.S., Abdul Kari Z., Pati S. (2022). A review on recent advances on natural plant pigments in foods: Functions, extraction, importance, and challenges. Appl. Biochem. Biotechnol..

[3] Sarmiento-Tovar A.A., Silva L., Sánchez-Suárez J., Diaz L. (2022). *Streptomyces* derived bioactive pigments: Ecofriendly source of bioactive compounds. Coatings.

[4] Law J.W.F., Letchumanan V., Tan L.T.H., Ser H.L., Goh B.H., Lee L.H. (2020). The rising of “modern actinobacteria” era. Prog. Microbes Mol. Biol..

[5] Bibb M.J. (2013). Understanding and manipulating antibiotic production in actinomycetes. Biochem. Soc. Trans..

[6] Dave A., Ingle S., Sayyed R., Singh A., Ilyas N. (2022). Potential of *Streptomyces* and its secondary metabolites for biocontrol of fungal plant pathogens. Antifungal Metabolites of Rhizobacteria for Sustainable Agriculture.

[7] Wynn-Williams D.D., Edwards H.G.M., Newton E.M., Holder J.M. (2002). Pigmentation as a survival strategy for ancient and modern photosynthetic microbes under high ultraviolet stress on planetary surfaces. Int. J. Astrobiol..

[8] Suresh M., Renugadevi B., Brammavidhya S., Iyapparaj P., Anantharaman P. (2015). Antibacterial activity of red pigment produced by *Halolactibacillus alkaliphilus* MSRD1-an isolate from seaweed. Appl. Biochem. Biotechnol..

[9] Djemouai N., Meklat A., Gaceb-Terrak R., Youcef K.O.H., Nacer A., Saadi S.A., Saad S., Verheecke-Vaessen C., Bouras N. (2022). *Streptomyces* species from the rhizosphere of the medicinal plant *Artemisia herba-alba Asso*: Screening for biological activities biological activities. Biologia.

[10] Peng F., Zhang M.Y., Hou S.Y., Chen J., Wu Y.Y., Zhang Y.X. (2020). Insights into *Streptomyces* spp. isolated from the rhizospheric soil of *Panax notoginseng*: Isolation, antimicrobial activity and biosynthetic potential for polyketides and non-ribosomal peptides. BMC Microbiol..

[11] Qi D., Zou L., Zhou D., Chen Y., Gao Z., Feng R., Zhang M., Li K., Xie J., Wang W. (2019). Taxonomy and Broad-Spectrum Antifungal Activity of *Streptomyces* sp. SCA3-4 Isolated from Rhizosphere Soil of *Opuntia stricta*. Front. Microbiol..

[12] Janković V., Marković D., Nikodinovic-Runic J., Radetić M., Ilic-Tomic T. (2023). Eco-friendly dyeing of polyamide and polyamide-elastane knits with living bacterial cultures of two *Streptomyces* sp. strains. World J. Microbiol. Biotechnol..

[13] Bystrykh L.V., Fernández-Moreno M.A., Herrema J.K., Malpartida F., Hopwood D.A., Dijkhuizen L. (1996). Production of actinorhodin-related “blue pigments” by *Streptomyces coelicolor* A3 (2). J. Bacteriol..

[14] Kramar A., Ilic-Tomic T., Petkovic M., Radulovic N., Kostic M., Jocic D., Nikodinovic-Runic J. (2014). Crude bacterial extracts of two new *Streptomyces* sp. isolates as bio-colorants for textile dyeing. World J. Microbiol. Biotechnol..

[15] El-Naggar N.E.A., El-Ewasy S.M. (2017). Bioproduction, characterization, anticancer and antioxidant activities of extracellular melanin pigment produced by newly isolated microbial cell factories *Streptomyces glaucescens* NEAE-H. Sci. Rep..

[16] Jumpathong J., Nuengchamnong N., Masin K., Nakaew N., Suphrom N. (2019). Thin layer chromatography-TLC-bioautographic assay for antibacterial compounds from *Streptomyces* sp. TBRC 8912, a newly isolated actinomycin D producer. Chiang Mai J. Sci..

[17] Charoenwiwattanakij P., Pratuangdejkul J., Chongruchiroj S., Suwanborirux K., Thawai C., Chingunpitak J., Satitpatipan V. (2020). Effect of actinomycin D isolated from the cultured broth of marine *Streptomyces* spp. on cell division protein FtsZ. Chiang Mai J. Sci..

[18] Kanchanasin P., Saeng-in P., Nakashima T., Matsuo H., Takahashi Y., Tanasupawat S. (2020). Actinomycins produced by *Streptomyces lichenis* LCR6-01^T^ and its antibacterial activity. Chiang Mai J. Sci..

[19] Qureshi K.A., Bholay A.D., Rai P.K., Mohammed H.A., Khan R.A., Azam F., Jaremko M., Emwas A.-H., Stefanowicz P., Waliczek M. (2021). Isolation, characterization, anti-MRSA evaluation, and in-silico multi-target anti-microbial validations of actinomycin X_2_ and actinomycin D produced by novel *Streptomyces smyrnaeus* UKAQ_23. Sci. Rep..

[20] Chen W., Ye K., Zhu X., Zhang H., Si R., Chen J., Chen Z., Song K., Yu Z., Han B. (2021). Actinomycin X_2_, an antimicrobial depsipeptide from marine-derived *Streptomyces cyaneofuscatus* applied as a good natural dye for silk fabric. Mar. Drugs.

[21] Shah A.M., Hussain A., Mushtaq S., Rather M.A., Shah A., Ahmad Z., Khan I.A., Bhat K.A., Hassan Q.P. (2017). Antimicrobial investigation of selected soil actinomycetes isolated from unexplored regions of Kashmir Himalayas, India. Microb. Pathog..

[22] Dong M., Cao P., Ma Y.T., Luo J., Yan Y., Li R.T., Huang S.X. (2019). A new actinomycin Z analogue with an additional oxygen bridge between chromophore and β-depsipentapeptide from *Streptomyces* sp. KIB-H714. Nat. Prod. Res..

[23] Sharma M., Manhas R.K. (2019). Purification and characterization of actinomycins from *Streptomyces* strain M7 active against methicillin resistant *Staphylococcus aureus* and vancomycin resistant *Enterococcus*. BMC Microbiol..

[24] Adlin Jenifer J.S.C., Michaelbabu M., Eswaramoorthy Thirumalaikumar C.L., Jeraldin Nisha S.R., Uma G., Citarasu T. (2019). Antimicrobial potential of haloalkaliphilic *Nocardiopsis* sp. AJ1 isolated from solar salterns in India. J. Basic Microbiol..

[25] Waksman S.A., Woodruff H.B. (1940). Bacteriostatic and bactericidal substances produced by a soil Actinomyces. Proc. Soc. Exp. Biol. Med..

[26] Singh S.B., Genilloud O., Peláez F. (2010). Terrestrial microorganisms–Filamentous bacteria. Compr. Nat. Prod. II.

[27] Farber S., D’Angio G., Evans A., Mitus A. (1960). Clinical studies on actinomycin D with special reference to Wilms’ tumor in children. Ann. N. Y. Acad. Sci..

[28] Bensaude O. (2011). Inhibiting eukaryotic transcription. Which compound to choose? How to evaluate its activity? Which compound to choose? How to evaluate its activity?. Transcription.

[29] Somphong A., Poengsungnoen V., Buaruang K., Sripreechasak P., Khantasup K., Intaraudom C., Pittayakhajonwut P., Tanasupawat S., Phongsopitanun W. (2023). The lichen-derived *Streptomyces* isolated from *Pyxine cocoes* produces the antibiotic with potent antimicrobial and antitumor activities. ScienceAsia.

[30] Chen C., Song F., Wang Q., Abdel-Mageed W.M., Guo H., Fu C., Hou W., Dai H., Liu X., Yang N. (2012). A marine-derived *Streptomyces* sp. MS449 produces high yield of actinomycin X_2_ and actinomycin D with potent anti-tuberculosis activity. Appl. Microbiol. Biotechnol..

[31] Machushynets N.V., Elsayed S.S., Du C., Siegler M.A., de la Cruz M., Genilloud O., Hankemeier T., van Wezel G.P. (2022). Discovery of actinomycin L, a new member of the actinomycin family of antibiotics. Sci. Rep..

[32] Zhou W., Xie Z., Si R., Chen Z., Javeed A., Li J., Wu Y., Han B. (2023). Actinomycin-X_2_-immobilized silk fibroin film with enhanced antimicrobial and wound healing activities. Int. J. Mol. Sci..

[33] Ahn S.-Y., Jang S., Sudheer P.D.V.N., Choi K.-Y. (2021). Microbial Production of Melanin Pigments from Caffeic Acid and L-Tyrosine Using *Streptomyces glaucescens* and FCS-ECH-Expressing *Escherichia coli*. Int. J. Mol. Sci..

[34] Zothanpuia P., Passari A., Singh B.P. (2015). Molecular characterization of actinomycetes isolated from Tuichang river and their biosynthetic potential. Sci. Vis..

[35] Geetanjali, Jain P. (2016). Antibiotic production by rhizospheric soil microflora-a review. Int. J. Pharm. Sci. Res..

[36] Wei Y., Zhao Y., Zhou D., Qi D., Li K., Tang W., Chen Y., Jing T., Zang X., Xie J. (2020). A newly isolated *Streptomyces* sp. YYS-7 with a broad-spectrum antifungal activity improves the banana plant resistance to *Fusarium oxysporum* f. sp. *cubense* tropical race 4. Front. Microbiol..

[37] Singh R., Dubey A.K. (2020). Isolation and characterization of a new endophytic actinobacterium *Streptomyces californicus* strain ADR1 as a promising source of anti-bacterial, anti-biofilm and antioxidant metabolites. Microorganisms.

[38] Mohseni M., Norouzi H., Hamedi J., Roohi A. (2013). Screening of antibacterial producing actinomycetes from sediments of the Caspian Sea. Int. J. Mol. Cell. Med..

[39] Robinson T., Singh D., Nigam P. (2002). Fermentación en estado sólido: Una tecnología microbiana promisoria para la producción de metabolitos secundarios. Vitae.

[40] Lee H.J., Han S.I., Whang K.S. (2012). *Streptomyces gramineus* sp. nov., an antibiotic-producing actinobacterium isolated from bamboo (*Sasa borealis*) rhizosphere soil. Int. J. Syst. Evol. Microbiol..

[41] Brockmann H., Pini H. (1947). Actinorhodin, ein roter Farbstoff aus Actinomyceten. Naturwissenschaften.

[42] Hemeda N.A., Hegazy G.E., Abdelgalil S.A., Soliman N.A., Abdel-Meguid D.I., El-Assar S.A. (2022). Maximization of red pigment production from *Streptomyces* sp. LS1 structure elucidation and application as antimicrobial/antifouling against human pathogens and marine microbes. J. Genet. Eng. Biotechnol..

[43] Mitchell A., Spencer M., Edmiston C. (2015). Role of healthcare apparel and other healthcare textiles in the transmission of pathogens: A review of the literature. J. Hosp. Infect..

[44] Colclasure V.J., Soderquist T.J., Lynch T., Schubert N., McCormick D.S., Urrutia E., Knickerbocker C., McCord D., Kavouras J.H. (2015). Coliform bacteria, fabrics, and the environment. Am. J. Infect. Control..

[45] Li N., Wang Q., Zhou J., Li S., Liu J., Chen H. (2022). Insight into the progress on natural dyes: Sources, structural features, health effects, challenges, and potential. Molecules.

[46] Kramar A., Kostic M.M. (2022). Bacterial secondary metabolites as biopigments for textile dyeing. Textiles.

[47] Ellaiah P., Srinivasulu B., Adinarayana K. (2004). Optimisation studies on neomycin production by a mutant strain of *Streptomyces marinensis* in solid state fermentation. Process Biochem..

[48] Liu H., Du X., Yuan Q., Zhu L. (2009). Optimisation of enzyme assisted extraction of silybin from the seeds of *Silybum marianum* by Box–Behnken experimental design. Phytochem. Anal. Int. J. Plant Chem. Biochem. Tech..

[49] Liu M., Jia Y., Xie Y., Zhang C., Ma J., Sun C., Ju J. (2019). Identification of the actinomycin D biosynthetic pathway from marine-derived *Streptomyces costaricanus* SCSIO ZS0073. Mar. Drugs.

[50] Wang D., Wang C., Gui P., Liu H., Khalaf S.M., Elsayed E.A., Wadaan M.A.M., Hozzein W.N., Zhu W. (2017). Identification, bioactivity, and productivity of actinomycins from the marine-derived *Streptomyces heliomycini*. Front. Microbiol..

[51] Wang M., Zhang Y., Wang R., Wang Z., Yang B., Kuang H. (2021). An evolving technology that integrates classical methods with continuous technological developments: Thin-layer chromatography bioautography. Molecules.

[52] Chandrakar S., Gupta A.K. (2019). Actinomycin-producing endophytic *Streptomyces parvulus* associated with root of *Aloe vera* and optimization of conditions for antibiotic production. Probiotics Antimicrob. Proteins.

[53] Kirk J.M. (1960). The mode of action of actinomycin D. Biochim. Biophys. Acta.

[54] Lee J.H., Kim Y.G., Lee K., Kim C.J., Park D.J., Ju Y., Lee J.C., Wood T.K., Lee J. (2016). *Streptomyces*-derived actinomycin D inhibits biofilm formation by *Staphylococcus aureus* and its hemolytic activity. Biofouling.

[55] Mu Y.Q., Xie T.T., Zeng H., Chen W., Wan C.X., Zhang L.L. (2020). *Streptomyces*-derived actinomycin D inhibits biofilm formation via downregulating ica locus and decreasing production of PIA in *Staphylococcus epidermidis*. J. Appl. Microbiol..

[56] Ramirez-Rodriguez L., Stepanian-Martinez B., Morales-Gonzalez M., Diaz L. (2018). Optimization of the cytotoxic activity of three *Streptomyces* strains isolated from Guaviare river sediments (Colombia, South America). BioMed Res. Int..

[57] Benbelkhir F.Z., Medjekal S. (2022). Microalgal carotenoids: A promising alternative to synthetic dyes. Algal Res..

[58] Venil C.K., Velmurugan P., Dufossé L., Devi P.R., Ravi A.V. (2020). Fungal pigments: Potential coloring compounds for wide ranging applications in textile dyeing. J. Fungi.

[59] Hamaki T., Suzuki M., Fudou R., Jojima Y., Kajiura T., Tabuchi A., Sen K., Shibai H. (2005). Isolation of novel bacteria and actinomycetes using soil-extract agar medium. J. Biosci. Bioeng..

[60] Lee L.H., Zainai N., Azman A.S., End S.K., Goh B.H., Yin W.F., Mutalib N.S., Chan K.G. (2014). Diversity and antimicrobial activities of actinobacteria isolated from tropical mangrove sediments in Malaysia. Sci. World J..

[61] Trusheva B., Trunkova D., Bankova V. (2007). Different extraction methods of biologically active components from propolis: A preliminary study. Chem. Cent. J..

[62] Lorian V., Lorian V. (1991). Laboratory methods used to assess the activity of antimicrobial combinations. Antibiotics in Laboratory Medicine.

[63] Hudzicki J. (2009). Kirby-Bauer disk diffusion susceptibility test protocol. Am. Soc. Microbiol..

[64] Weisburg W.G., Barns S.M., Pelletier D.A., Lane D.J. (1991). 16S ribosomal DNA amplification for phylogenetic study. J. Bacteriol..

[65] Tamura K., Stecher G., Kumar S. (2021). MEGA11: Molecular evolutionary genetics analysis version 11. Mol. Biol. Evol..

[66] Jeyaseelan E.C., Jenothiny S., Pathmanathan M.K., Jeyadevan J.P. (2012). Antibacterial activity of sequentially extracted organic solvent extracts of fruits, flowers and leaves of *Lawsonia inermis* L. from Jaffna. Asian Pac. J. Trop. Biomed..

[67] Ho Y., Suphrom N., Daowtak K., Potup P., Thongsri Y., Usuwanthim K. (2020). Anticancer Effect of *Citrus hystrix* DC. Leaf Extract and Its Bioactive Constituents Citronellol and, Citronellal on the Triple Negative Breast Cancer MDA-MB-231 Cell Line. Pharmaceuticals.

[68] Dewanjee S., Gangopadhyay M., Bhattacharya N., Khanra R., Dua T.K. (2015). TLC-bioautographic assay and its scope in the field of natural product chemistry. J. Pharm. Anal..

[69] Yamaç M., Bilgili F. (2006). Antimicrobial activities of fruit bodies and/or mycelial cultures of some mushroom isolates. Pharm. Biol..

[70] Liu C.W., Lu Y.Y., Yang Z.Z., Xing Y.Y., Xi T. (2010). Rapid screening and characterization of metabolites from a marine-derived actinomycete by high-performance liquid chromatography coupled with electrospray ionization quadrupole time-of-flight mass spectrometry. Rapid Commun. Mass Spectrom..

[71] (2011). Parallel Streak: Assess.

[72] (2019). Test Method for Antibacterial Finishes on Textile Materials: Assess.

[73] Sriwiriyajan S., Ninpesh T., Sukpondma Y., Nasomyon T., Graidist P. (2014). Cytotoxicity screening of plants of genus *Piper* in breast cancer cell lines. Trop. J. Pharm. Res..

