# Supplementary material for: Actinomycins from Soil-Inhabiting Streptomyces as Sources of Antibacterial Pigments for Silk Dyeing
Source: Molecules. 2023 Aug 8;28(16):5949. doi: 10.3390/molecules28165949 (PMC10459128; doi:10.3390/molecules28165949)

Supplementary data

Table S1. Detail of *Streptomyces* isolated from soils and accession numbers in GenBank.

Culture code	NCBI Accession Number	Identification of actinobacteria	Plant taxa where soil was collected
TBRC 15924	ON406138	*Streptomyces* sp.	*Magnolia baillonii*
TBRC 15925	ON406139	*Streptomyces shenzhenensis*	*Magnolia rajaniana*
TBRC 15926	ON406140	*Streptomyces* sp.	*Magnolia rajaniana*
TBRC 15927	ON406143	*Streptomyces gramineus*	*Magnolia baillonii*
TBRC 15928	ON406144	*Streptomyces* sp.	*Magnolia rajaniana*
TBRC 15929	ON406141	*Streptomyces adustus*	*Cinnamomum parthenoxylon*
TBRC 15930	ON406145	*Streptomyces* sp.	*Magnolia rajaniana*
TBRC 15931	ON406142	*Streptomyces gramineus*	*Magnolia baillonii*

Table S2. Fractionation using silica gel column chromatography no. 1 column (size 33.5 × 3 cm 3,000 mg)

No.	Fractionation	Elution system	Weight (mg)
1	G.1	Dichloromethane	68.1
2	G.2	Dichloromethane	804.7
3	G.3	Dichloromethane	20.4
4	G.4	Dichloromethane	13.8
5	G.5	Dichloromethane	16
6	G.6	Dichloromethane	13.9
7	G.7	Dichloromethane	100.1
8	G.8	5% Methanol/ Dichloromethane	1178.6
9	G.9	5% Methanol/ Dichloromethane	13.4
10	G.10	5% Methanol/ Dichloromethane	6.5
11	G.11	5% Methanol/ Dichloromethane	6.6
12	G.12	5% Methanol/ Dichloromethane	36.8
13	G.13	5% Methanol/ Dichloromethane	8.2
14	G.14	7% Methanol/ Dichloromethane	1.8
15	G.15	7% Methanol/ Dichloromethane	25.5
16	G.16	10% Methanol/ Dichloromethane	30.7
17	G.17	10% Methanol/ Dichloromethane	7.8
18	G.18	10% Methanol/ Dichloromethane	7.3
19	G.19	10% Methanol/ Dichloromethane	4.8
20	G.20	10% Methanol/ Dichloromethane	3.9
21	G.21	10% Methanol/ Dichloromethane	6.6
22	G.22	10% Methanol/ Dichloromethane	1.8
23	G.23	20% Methanol/ Dichloromethane	62.5
24	G.24	30% Methanol/ Dichloromethane	125.2
25	G.25	40-70% Methanol/ Dichloromethane	77.5
26	G.26	80% Methanol/ Dichloromethane	72.2
		Total	2714.7

Table S3. Fractionation using silica gel column chromatography no. 2 column (size 23.5 × 1.5 cm, 500 mg)

No.	Fractionation	Elution system	Weight (mg)
1	SG.1	Chloroform	0.8
2	SG.2	Chloroform	2.2
3	SG.3	Chloroform	9.6
4	SG.4	Chloroform	49.7
5	SG.5	Chloroform	16.7
6	SG.6	Chloroform	15.9
7	SG.7	Chloroform	13.5
8	SG.8	Chloroform	10.4
9	SG.9	Chloroform	18.7
10	SG.10	Chloroform	8
11	SG.11	Chloroform	1.8
12	SG.12	Chloroform	10.6
13	SG.13	Chloroform	0.6
14	SG.14	Chloroform	2.6
15	SG.15	Chloroform	1.1
16	SG.16	Chloroform	21.4
17	SG.17	Chloroform	2.3
18	SG.18	Chloroform	0.8
19	SG.19	Chloroform	2.2
20	SG.20	Chloroform	7.5
21	SG.21	0.5% Acetone/Chloroform	1.4
22	SG.22	0.5% Acetone/Chloroform	5.7
23	SG.23	0.5% Acetone/Chloroform	22.4
24	SG.24	0.5% Acetone/Chloroform	25.7
25	SG.25	1% Acetone/Chloroform	19.7
26	SG.26	1% Acetone/Chloroform	31.9
27	SG.27	2% Acetone/Chloroform	47.8
28	SG.28	10% Acetone/Chloroform	16.2
29	SG.29	10% Acetone/Chloroform	8.7
30	SG.30	50% Acetone/Chloroform	8
31	SG.31	50% Acetone/Chloroform	6.1
32	SG.32	50% Acetone/Chloroform	3.1
33	SG.33	100% Acetone	1.5
34	SG.34	100% Acetone	3.9
35	SG.35	100% Acetone	2.6
36	SG.36	100% Methanol	11.7
37	SG.37	100% Methanol	2.9
		Total	415.7

Table S4 Solvent systems (% v/v) for dissolving the selected fractions and for TLC development

Fraction no.	Solvent system for sample dissolving	Solvent system (mobile phase) for TLC development
f.10	50% dichloromethane in methanol	50% chloroform in acetone
f.22	50% dichloromethane in methanol	5% methanol in dichloromethane
f.23	50% dichloromethane in methanol	3% methanol in dichloromethane
f.24	50% dichloromethane in methanol	3% methanol in dichloromethane
f.25	50% dichloromethane in methanol	3% methanol in dichloromethane
f.26	50% dichloromethane in methanol	3% methanol in dichloromethane
f.27	50% dichloromethane in methanol	5% methanol in dichloromethane
f.28	50% dichloromethane in methanol	7% methanol in dichloromethane
f.29	50% dichloromethane in methanol	7% methanol in dichloromethane
f.30	50% dichloromethane in methanol	7% methanol in dichloromethane
f.31	50% dichloromethane in methanol	7% methanol in dichloromethane
f.36	methanol	10% methanol in dichloromethane

Figure S1 QTOF MS/MS spectra of actinomycins at fragmentor voltage of CID@40.0: A) Actinomycin X_2 detected in the crude ethyl acetate extract of TBRC 15931 (code TN166); B) Sample of standard actinomycin X_2.

A

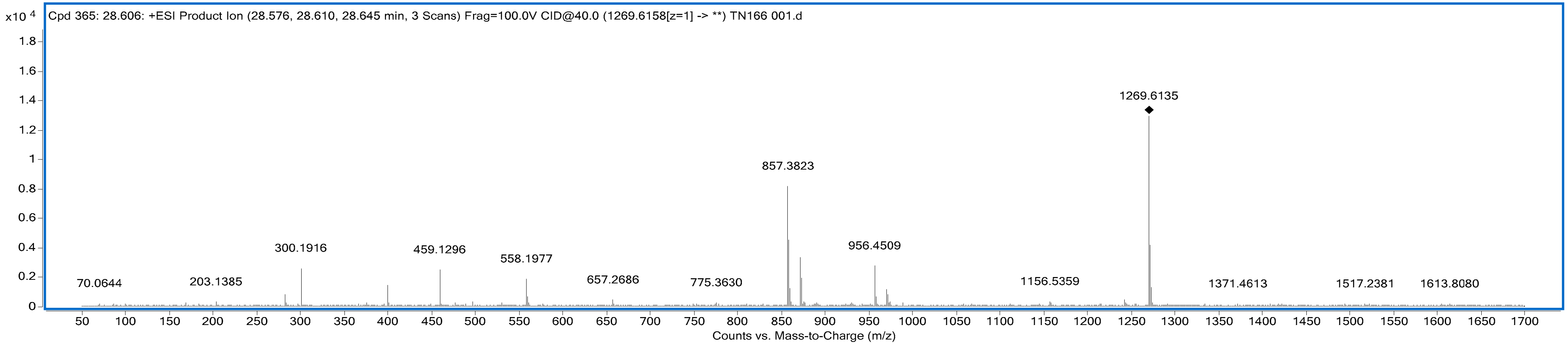

B

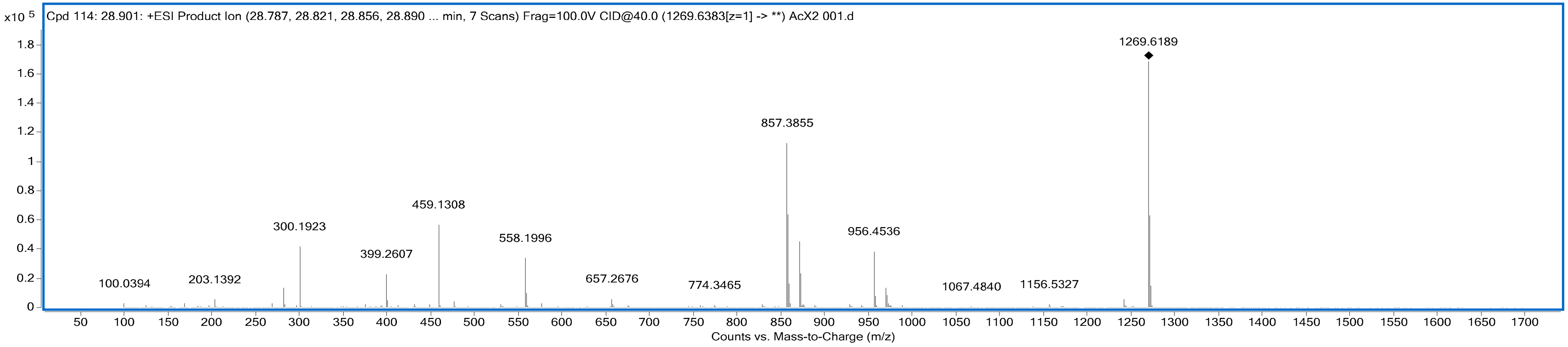

Supplement: Supplementary file 1 [file molecules-28-05949-s001.zip › molecules-2493391-supplementary.pdf]
